# Probiotic supplementation mitigates sex-dependent nociceptive changes and gut dysbiosis induced by prenatal opioid exposure

**DOI:** 10.1080/19490976.2025.2464942

**Published:** 2025-02-14

**Authors:** Salma Singh, Yaa Abu, Danielle Antoine, Daniel Gomez, Junyi Tao, Bridget Truitt, Sabita Roy

**Affiliations:** a Department of Surgery, School of Medicine, University of Miami Miller, Miami, USA; b Department of Neuroscience, School of Medicine, University of Miami Miller, Miami, USA

**Keywords:** Prenatal opioid exposure, gut microbiome, dysbiosis, sex-based differences, nociception

## Abstract

The gut microbiome has emerged as a promising target for modulating adverse effects of opioid exposure due to its significant role in health and disease. Opioid use disorder (OUD) has become increasingly prevalent, specifically in women of reproductive age, contributing to an increased incidence of offspring exposed to opioids in utero. Recent studies have shown that prenatal opioid exposure (POE) is associated with notable changes to the maternal gut microbiome, with subsequent implications for the offspring’s microbiome and other adverse outcomes. However, the role of the gut microbiome in mediating sex-based differences in pain sensitivity has not yet been investigated. In this study, both male and female C57BL/6 offspring were used to determine sex-based differences in nociception and gut microbial composition as a result of POE. Our data reveals significant sex-based differences in offspring prenatally exposed to opioids. The gut microbiome of opioid-exposed females showed an enrichment of commensal bacteria including *Lactobacillus* compared to opioid-exposed males. Additionally, POE females demonstrated decreased nociceptive sensitivity, while males demonstrated increased nociceptive sensitivity. RNA sequencing of the prefrontal cortex showed sex-based differences in several canonical pathways, including an increase in the opioid signaling pathway of opioid-exposed females, which was not observed in males. Microbiome modification via maternal probiotic supplementation attenuated sex-based differences throughout the early stages of life. Together, our study provides further insight on sex-based differences arising from POE and highlights the pivotal role of the gut microbiome as a modifiable target for mitigating its negative effects.

## Introduction

Opioid use in the United States has surged, especially among women of reproductive age.^1^ Consequently, more infants are exposed to opioids in utero, which can result in neonatal opioid withdrawal syndrome (NOWS) after birth due to placental opioid transfer. Pregnant women with opioid dependence are often prescribed opioid agonists, including methadone or buprenorphine, to manage opioid use disorder (OUD) and reduce the risk of NOWS.^2^ However, these treatments remain controversial due to their association with adverse outcomes^3^and their classification as prenatal opioid exposure (POE). Despite POE’s high prevalence, its effects on fetal development and postnatal outcomes remain unclear. Nevertheless, recent studies suggest POE influences behavioral and developmental outcomes,^4^ with the gut microbiome playing a significant role.^5^

The gut microbiome, composed of a variety of bacteria and fungi, plays a key role in maintaining health by regulating immune responses^6^ and communicating with the brain via the gut-brain axis.^7^ Dysbiosis, or imbalance in gut microbial composition, is observed in neurological conditions, including depression, neuropathic pain, and opioid tolerance.^8^ Opioid receptors are widely distributed throughout the body, particularly in the brain and gut, and are activated by numerous endogenous and exogenous substances, including opioids.^9^ Consequently, opioid use has been shown to induce gut dysbiosis and compromise the integrity of the intestinal barrier,^10^ increasing the host’s susceptibility to various diseases.

Studies have demonstrated that even brief exposure to opioids during pregnancy can result in maternal gut dysbiosis, which subsequently affects the balance of microbial communities in offspring.^11^ Additionally, our recent study involving 3-week-old male offspring prenatally exposed to opioids highlights the potential role of the gut-brain axis in mediating nociception.^5^ These male offspring prenatally exposed to opioids showed altered gut microbial composition, heightened sensitivity to thermal pain, and transcriptional alterations in genes related to several pathways including neuropathic pain signaling and neuroinflammation. Maternal probiotic supplementation mitigated the adverse outcomes observed post-exposure, with FMT studies further demonstrating the gut microbiome’s role in male offspring. However, data on female offspring is lacking.

There is accumulating evidence that sex plays a significant role in modulating response to acute stimuli as well as adaptation to chronic stimuli,^12,13^ with women generally showing greater pain sensitivity using subjective^14,15^ and objective metrics.^16,17^ However, some studies have not identified consistent patterns of sex-based differences in humans.^18^ While human pain involves a complex interplay of biological, psychological, and sociocultural factors, animal models – particularly murine models – have been instrumental in elucidating POE’s effects on brain development and nociception.^4,19–21^ Unfortunately, many studies have exclusively used male animals, limiting the external validity.^22^ To date, sex-based differences in thermal pain sensitivity following POE have not yet been described.

The mechanism(s) underlying sex differences in opioid exposure are likely multifactorial^23–25^ and remain elusive. Three opioid receptors—μ, κ, and δ—have been implicated in pain modulation,^26^ with several studies proposing that μ-opioid receptors may exhibit increased potency in females.^27,28^ Studies have also uncovered sex differences in drug disposition,^29^ though others have found no significant difference in serum drug levels.^30^ Additional contributing factors may include the impact of endogenous opioid peptides on morphine potency^31^ and the modulation of analgesics by sex hormones.^32,33^ Ongoing work aims to define sex-based effects on developmental responses to various prenatal exposures, including obesity risk,^34^ drugs of abuse,^25,35^ and the role of genetics in pain modulation.^36,37^

This current study aims to uncover sex-specific differences in gut microbiome composition, nociception, and to identify transcriptional changes following POE in a longitudinal manner. Given the well-established role of the gut microbiome in health and disease, and its bidirectional communication with the brain via the gut-brain axis, the gut microbiome represents a plausible target for mitigating the effects of POE. Here, we hypothesize that modulation of the maternal microbiome through the administration of a probiotic formulation composed of specific commensal bacterial strains can attenuate the negative developmental outcomes associated with POE. This approach offers a potential therapeutic strategy to counteract the long-term impact of POE on neurodevelopment and nociception.

## Methods and materials

### Animals

Female and male C57BL/6 mice (10–12 weeks old) were obtained from The Jackson Laboratory and housed in groups of up to five per cage. The mice were kept in individually ventilated cages within a barrier facility maintained on a 12-hour light/12-hour dark cycle at a constant temperature (72 ± 1 °F) and humidity level (50%). All experimental procedures were conducted during the light-cycle. Mice were randomly assigned to experimental groups and provided with the same diet and living conditions, with food and water available *ad libitum*. Animal studies were conducted in accordance with protocols set forth by the University of Miami Institutional Animal Care and Use Committee (IACUC) and in compliance with rules and guidelines described by the National Institute of Health Guide for the Care and Use of Laboratory Animals.

### Experimental design

Fourteen days prior to mating, healthy female C57BL/6 mice were randomly assigned to receive increasing doses of hydromorphone (b.i.d., sc.) to induce opioid dependence. The dosing regimen was as follows: 0.5 mg/kg during pre-gestational days 1–3, 1.25 mg/kg during pre-gestational days 4–6, 2 mg/kg during pre-gestational days 7–9, 2.75 mg/kg during pre-gestational days 10–12, and 3.5 mg/kg during pre-gestational days 13–14. Subsequently, the females were mated with age-matched, healthy males at a 2:1 ratio. At this point, female mice began treatment with methadone (10 mg/kg, b.i.d., sc.), as opioid maintenance therapy drug, where 8–16 mg/kg is considered clinically relevant.^38^ Methadone treatment continued throughout the gestational period until weaning (postnatal day 21). Control animals were administered saline within the same timeline.

**Figure 1.**
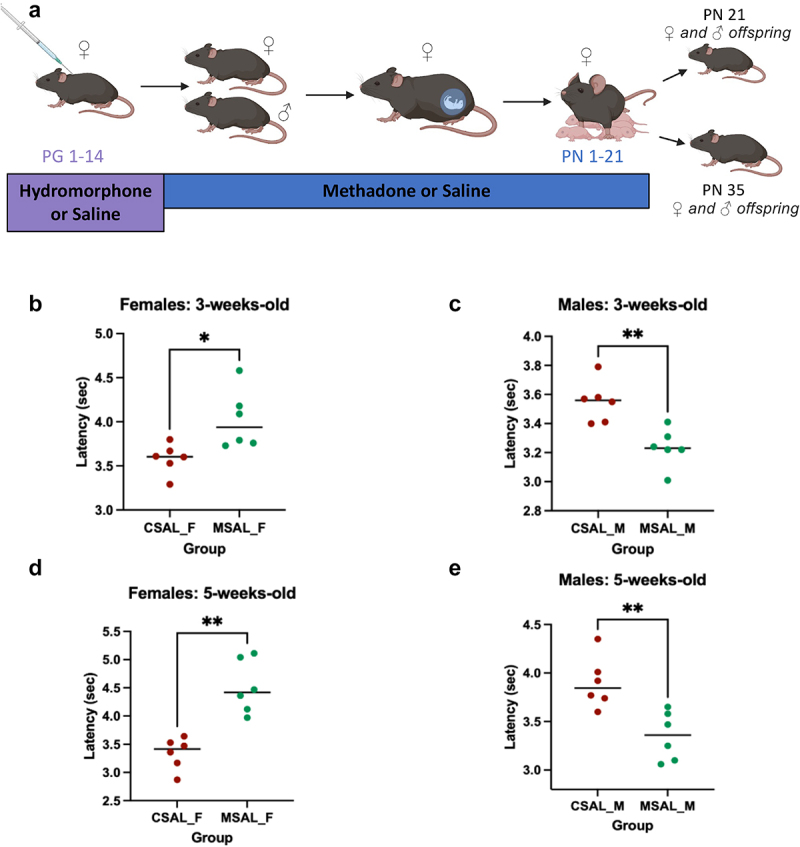
Animal schematic and thermal pain sensitivity of offspring prenatally exposed to saline (CSAL) or opioids (MSAL). (a) From PG1–14, female C57BL/6 mice were administered an escalating dose of hydromorphone to induce opioid dependence. Afterwards, females were mated with drug naïve, age-matched males and transitioned to methadone; treatments persisted through PN1–21. Tail flick latencies of the offspring was assessed at 3-weeks-old and 5-weeks old. (b) Tail flick latency of 3-week-old female offspring (*n* = 6 per group). (c) Tail flick latency of 3-week-old male offspring (*n* = 6 per group). (d) Tail flick latency of 5-week-old female offspring (*n* = 6 per group). (e) Tail flick latency of 5-week-old male offspring (*n* = 6 per group).

Once pregnant, dams were housed with a maximum of two per cage. Approximately 3–5 days prior to birth, dams were randomly assigned to be gavaged with either VSL#3, serving as a probiotic treatment, or with water as a control, until weaning. VSL#3 is composed of eight bacterial strains (four *Lactobacillus*, three *Bifidobacterium*, and one *Streptococcus*) and prepackaged at a concentration of 450 billion colony forming units (CFU) per packet. Daily, each packet was diluted in sterile water to its desired concentration, then each animal of the respective experimental group was given 0.2 mL. To verify the concentration, cultural analyses was completed using retrospective plate counts on blood agar plates. After birth, at the weaning age of 3-weeks-old, the offspring were separated from the dams and housed based on sex and treatment, with a maximum of five mice per cage. Thermal pain sensitivity was evaluated in 3-week-old offspring at weaning and, in longitudinal studies, at 5-weeks-old (Figures 1a and 4). Mice were sacrificed using a CO_2_ inhalation as the primary method of euthanasia, followed by cardiac puncture. Post-mortem samples were collected aseptically and stored at −80°C.

*p < 0.05; **p ≤ 0.0099

Abbreviations: PG: Pre-gestation; PN: Post-natal

### Thermal pain

Nociception of all offspring prenatally exposed to their respective treatments was assessed using the tail-flick assay (Columbia Instruments, Columbus, OH) at 3 and 5 weeks of age (Supplemental Figure 1). On the day of testing, mice were placed into a restrainer and positioned on the tail-flick apparatus, with their tails placed in a groove exposed to a high-intensity light beam that generated a heat sensation upon activation. When the mice perceived the heat as painful, they rapidly withdrew their tails, and the latency to withdrawal was recorded. The heat intensity was set such that control animals exhibited an average latency between 3 and 5 seconds. Higher latency times indicated hyposensitivity, while lower latency times indicated hypersensitivity to thermal pain. A cutoff time of 10 seconds was used to prevent tissue damage. The restrainer and tail-flick apparatus were thoroughly cleaned between each mouse.

### Gut microbiome analysis

At the time of sacrifice, fecal contents from the colon were collected and stored at a temperature of −80°C until processing. Genomic DNA isolation was completed for further analysis using DNeasy PowerSoil Pro QIAcube HT Kit from Qiagen along with QIAcube HT liquid-handling machine (Qiagen, Maryland, USA). 16S amplification and sequencing were completed by the University of Minnesota Genomic Center. The hypervariable V4 region of the 16S rRNA gene was investigated and was PCR amplified using the forward primer 515F (GTGCCAFCMGCCGCGGTAA) and reverse primer 806 R (GGACTACHVGGGTWTCTAAT) to produce 427 base pair (bp) amplicons. The Illumina MiSeq v.3 platform was used to sequence the amplicons, generating 300-bp paired-end reads.

Sequence reads were clustered into amplicon sequence variants (ASVs) with the DADA2 package (version 1.26.0).^39^ With the DADA2 pipeline, error filtering, trimming, learning of error rates, denoising, merging of paired reads, and removal of chimeras were performed. The filtering parameters used were: maxN = 0, truncQ = 2, rm.phix=TRUE and maxEE = 2. For paired end reads truncLen 200, 190 was used to overlap after truncation to merge for forward and reverse reads, respectively. Error rate was obtained using the parametric error model (err) and these error rates were used to infer unique reads from the samples. Merging of the paired reads was done by aligning the denoised forward reads with the reverse-complement of the corresponding denoised reverse reads and constructing the merged “contig” sequences. An amplicon sequence variant (ASV) table and a higher-resolution version of the OTU table was constructed with merged data frames using traditional methods. Chimeric ASVs were removed, and taxonomy assignment was done using function assign. Taxonomy to the SILVA v138 reference database including species with 99% similarity and for species exact match was performed to improve accuracy. To generate alpha and beta diversity plots, taxonomy bar plots and linear discriminant analysis effect size (LEfSe) plotASV and taxonomy tables were imported in MicrobiomeAnalyst.^40^ A minimum count of 4 and prevalence filter 20% was set for low count filter and ASVs with less than 10% variation measured by inter-quantile range were also filtered. The data was normalized with total sum scaling and the threshold on the logarithmic LDA score for discriminative features was set to 2.

### RNA sequencing

Brain removal was completed aseptically at the time of sacrifice and separated into the left and right hemispheres. The left hemisphere was collected without further dissection and the right hemisphere was further dissected into several regions, including the prefrontal cortex, midbrain, and hippocampus. Each sample was stored individually at a temperature of −80°C. RNA sequencing was completed on the prefrontal cortex of the right brain by Novogene.

### Differential expressed genes (DEG) screening and pathway enrichment analysis

Differentially expressed genes (DEGs) between pairwise groups were calculated using the DESeq2 package (ver. 1.38.2) with R version 4.2.2 (2022-10-31). Using the Benjamini and Hochberg approach, *p* values were adjusted to control false discovery rates (FDRs). The threshold for significant DEGs were set to adjusted *p* < 0.05, and log2 fold change ≥ 0.5. DEGs were analyzed with the QIAGEN Ingenuity Pathway Analysis (IPA) to predict functional annotations, canonical pathways. The threshold of DEGs was set to |log2FoldChange| ≥0.5 & *p*-value <0.05. Fisher’s exact test was used to identify the overrepresented proteins or genes with a p-value of less than 0.05. Canonical pathways associated with different neurotransmitter systems as well as other functions, including behavior, nervous system development and function, cell – cell signaling, and cellular assembly and organization were identified.

### Statistical analysis

To analyze for statistical significance, Two-way ANOVA was performed using GraphPad Prism Version 9.5.1. Asterisks were used to represent statistical significance, and further described in figure descriptions. To analyze α-diversity, the Mann-Whitney or Kruskal-Wallis’s test were used. To analyze β-diversity, permutational multivariate analysis of variance (PERMANOVA) was used to determine statistical significance or lack thereof.

## Results

### Sex-based differences in thermal pain sensitivity

Several studies have found that prenatal exposure to buprenorphine or methadone increases pain sensitivity.^21,41^ However, limited research has focused on whether these findings are sex-based and/or longitudinal. Following the prenatal treatments (Figure 1a), the nociceptive thresholds of male and female offspring were assessed using the tail-flick assay. At 3 weeks of age, POE female offspring (MSAL_F) displayed a higher thermal pain threshold compared to those prenatally exposed to saline (CSAL_F), indicating hyposensitivity to pain (Figure 1b). In contrast, POE male offspring (MSAL_M) at 3 weeks displayed lower tail-flick latencies, indicating significant thermal pain hypersensitivity relative to saline-exposed animals (CSAL_M) (Figure 1c).

To assess the longitudinal effects of POE, nociceptive thresholds were also evaluated at 5 weeks of age. MSAL_F offspring at 5-weeks-old continued to exhibit significant thermal pain hyposensitivity compared to CSAL_F (Figure 1d), while MSAL_M continued to show hypersensitivity to thermal pain relative to CSAL_M (Figure 1e). Together, these data suggest persistent hyposensitivity to thermal pain in female offspring and hypersensitivity in male offspring prenatally exposed to opioids, indicating sex-based differences in nociception.

### Sex-based differences in gut microbiome composition

The gut microbiome has been found to significantly impact health and disease states, as well as pain perception. For instance, some studies have suggested that specific strains of *Lactobacillus*, a known commensal bacterium, influence the expression of μ-opioid receptors and promote analgesia,^42^ while others have identified its role in modulating visceral pain.^43^ In previously published work, we demonstrated the role of the gut microbiome in influencing pain sensitivity in POE male offspring through FMT.^5^ Building on this, we now examine gut microbial composition in both female and male offspring prenatally exposed to methadone using 16s rRNA sequencing.

**Figure 2.**
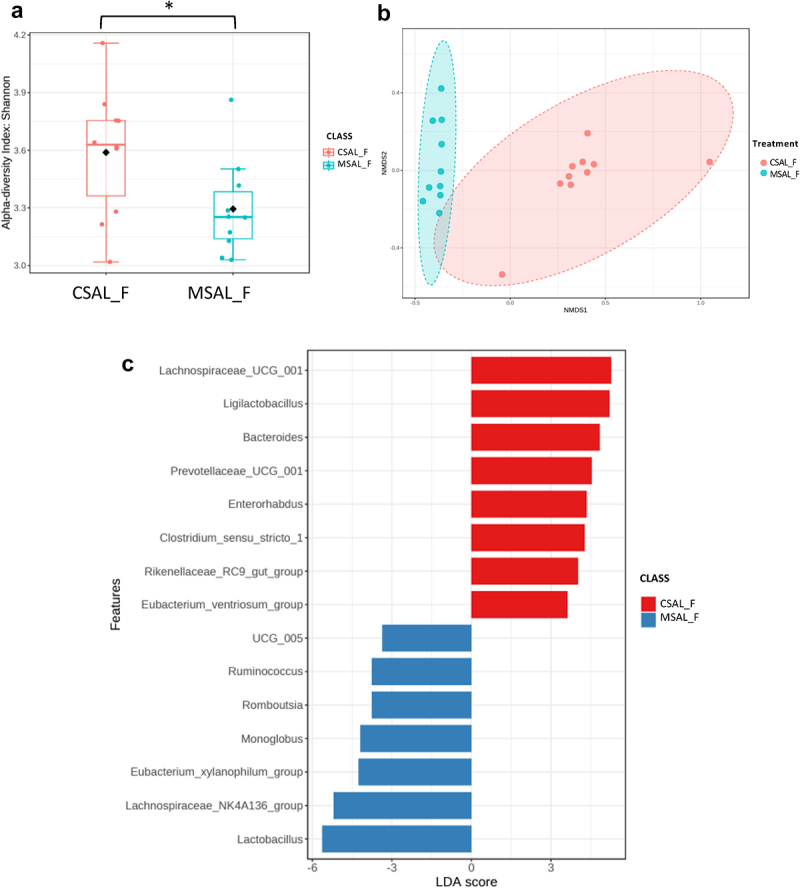
Microbiome analysis of female offspring (CSAL_F vs. MSAL_F) (*n* = 10 per group in all experiments). (a) α-diversity index represented using Shannon metric demonstrated significant differences in microbial richness between POE female offspring and respective controls. (b) β-diversity analysis represented using the Bray-Curtis metric indicated distinct clustering in the microbial composition between POE female offspring and their respective controls (*p* = 0.001). (c) LEfSe analysis of bacterial taxa at the genus level.

**Figure 3.**
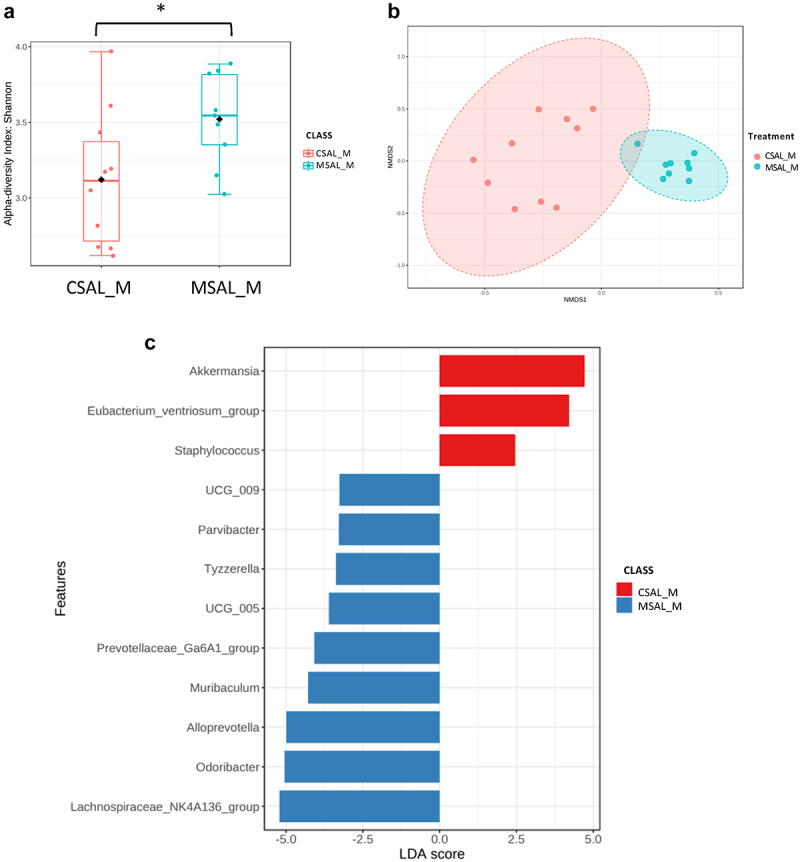
Microbiome analysis of male offspring (CSAL_M vs. MSAL_M) (*n* = 10 per group in all experiments). (a) α-diversity index represented using Shannon metric demonstrated significant differences in microbial richness between POE male offspring and respective controls. (b) β-diversity analysis represented using the Bray-Curtis metric indicated distinct clustering in the microbial composition between POE male offspring and their respective controls (*p* = 0.001). (c) LEfSe analysis of bacterial taxa at the genus level.

Fecal samples were collected from 5-week-old offspring, and microbiome analysis was performed on samples obtained from the distal colon. Significant sex-based differences in microbiome composition were observed. In comparing CSAL_F and MSAL_F offspring, significant differences were detected in α-diversity using the Shannon metric (Figure 2a) and in β-diversity (Figure 2b). Bacterial taxa such as *Ligilactobacillus* and *Bacteriodes* were abundant in CSAL_F, whereas *Lactobacillus* and *Ruminococcus* were abundant in MSAL_F offspring (Figure 2c). Male offspring prenatally exposed to opioids (MSAL_M) exhibited increased α-diversity (Figure 3a) and significantly different β-diversity (Figure 3b) compared to controls (CSAL_M). In MSAL_M offspring, an abundance of several bacterial taxa including *Parvibacter, Tyzzerella*, and *Alloprevotella* was observed, while taxa such as *Akkermansia* and *Staphylococcus* were more abundant in CSAL_M (Figure 3c). These data suggest that the microbiome of both female and male offspring prenatally exposed to opioids differs from their control counterparts, with several bacterial taxa found to be differentially enriched.

*p < 0.05.

*p < 0.05.

### Maternal probiotic intervention normalizes sex-based differences in pain sensitivity

**Figure 4.**
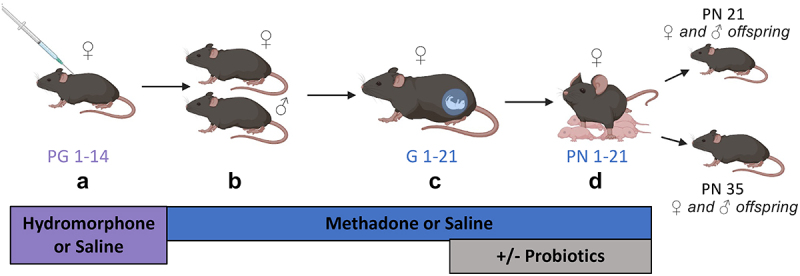
Mouse schematic of maternal probiotic intervention. (a) Throughout PG 1–14, female C57BL/6 mice were administered an escalating dose of hydromorphone for 14 days to induce opioid dependence. (b) Female C57BL/6 mice were mated with drug naïve, age-matched males and transitioned to methadone. (c) Throughout pregnancy, dams continued their respective treatments. 3–5 days prior to birth, they were randomly assigned to probiotic or water oral treatment. (d) All methadone/saline and probiotic/water treatments persisted after birth and until weaning (PN21). (e) Behavioral testing was completed at 3-weeks-old and 5-weeks old. Sacrifice was completed at 5-weeks-old.

The gut microbiome has been identified as a therapeutic target in pain sensitivity and various other conditions.^5,42^ Our previous study showed that male offspring prenatally exposed to methadone displayed hypersensitivity to thermal pain compared to saline-exposed counterparts, which could be normalized by administration of VSL#3 probiotics to mothers during pregnancy.^5^ We also demonstrated that maternal probiotic supplementation during pregnancy was sufficient to alter both the maternal and neonatal microbiome of male offspring. However, it is unclear how this affects the microbiome of female offspring or whether sex-based differences exist. Accordingly, this probiotic solution, consisting of several bacterial strains, was used to supplement the maternal gut microbiome with more commensal bacteria, promoting its homeostatic and mutually beneficial relationship with the host. Probiotic intervention was initiated in pregnant mothers 3–5 days prior to delivery and continued throughout weaning to modify the maternal microbiome and induce similar effects in the offspring microbiome (Figure 4).

**Figure 5.**
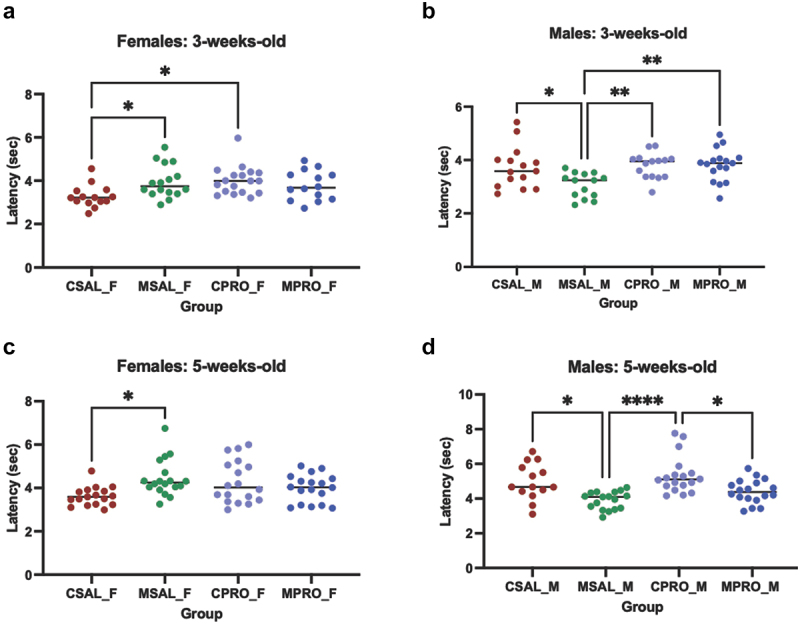
Thermal pain sensitivity of offspring prenatally exposed to saline or opioids at 3- or 5-weeks. (a) Tail flick latency of 3-week-old female offspring (CSAL_F *n* = 14; MSAL_F *n* = 16; CPRO_F *n* = 18; MPRO_F *n* = 14). (b) Tail flick latency of 3-week-old male offspring (CSAL_M *n* = 15; MSAL_M *n* = 14; CPRO_M *n* = 15; MPRO_M *n* = 17). (c) Tail flick latency of 5-week-old female offspring (CSAL_F *n* = 17; MSAL_F *n* = 18; CPRO_F *n* = 18; MPRO_F *n* = 18). (d) Tail flick latency of 5-week-old male offspring (CSAL_M *n* = 14; MSAL_M *n* = 17; CPRO_M *n* = 18; MPRO_M *n* = 18).

Interestingly, our data showed that with this probiotic intervention, 3-week-old female offspring exposed to both opioids and probiotics (MPRO_F) displayed no significant difference in thermal threshold compared to those exposed to saline and probiotics (CPRO_F) (Figure 5a). However, the probiotic intervention significantly increased the thermal threshold of control offspring (CPRO_F), reversing the observed hyposensitivity to pain in female offspring (CSAL_F v MSAL_F) (Figure 5a). In 3-week-old male offspring, probiotic intervention abolished hypersensitivity to thermal pain demonstrated by no significant differences observed between offspring (CPRO_M v MPRO_M) as observed between CSAL_M and MSAL_M. Probiotic intervention increased the thermal threshold of MPRO_M causing opioid and probiotic exposed offspring to be significantly different from opioids alone (MSAL_M), while not significantly different to CPRO_M. Both probiotic-exposed groups (CPRO_M and MPRO_M) had significantly higher latency times compared to those exposed to opioids alone (MSAL_M) (Figure 5b). However, probiotic supplementation did not appear to affect male offspring prenatally exposed to saline (CPRO_M v CSAL_M) (Figure 5b).

*p < 0.05; **p ≤ 0.0099; ****p ≤ 0.0001.

To determine how long the effect of maternal probiotic supplementation persists, tail flick experiments were conducted at 5-weeks-old. In 5-week-old female offspring whose mothers had received probiotics, no significant difference in tail flick latency was observed between those exposed to opioids or saline (CPRO_F v MPRO_F) (Figure 5c). However, the increased tail-flick latency between CSAL_F and CPRO_F was no longer observed (Figure 5c). Male offspring at 5 weeks showed different trends. MPRO_M was no longer significantly different from opioid-exposed male offspring (MSAL_M), but now displayed hypersensitivity to thermal pain relative to CPRO_M (Figure 5d). However, the tail-flick latency of MPRO_M continued to show no significant difference compared to CSAL_M (Figure 5d). Altogether, the data suggests that probiotics impact thermal sensitivity in a sex-dependent manner that changes with age.

**Figure 6.**
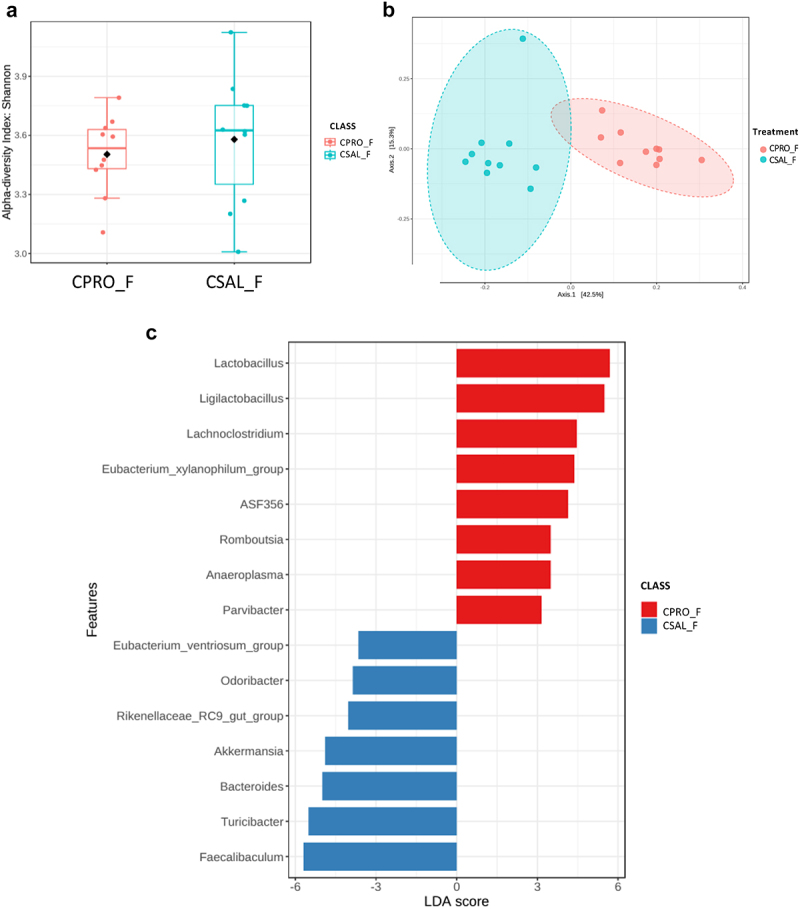
Microbiome analysis of control female offspring with or without prenatal exposure to probiotics (CPRO_F vs CSAL_F) (*n* = 10 per group in all experiments). (a) α-diversity index represented using Shannon metric demonstrated no significant differences in microbial richness. (b) β-diversity analysis represented using the Bray-Curtis metric indicated distinct clustering in the microbial composition (*p* = 0.001). (c) LEfSe analysis of bacterial taxa at the genus level.

**Figure 7.**
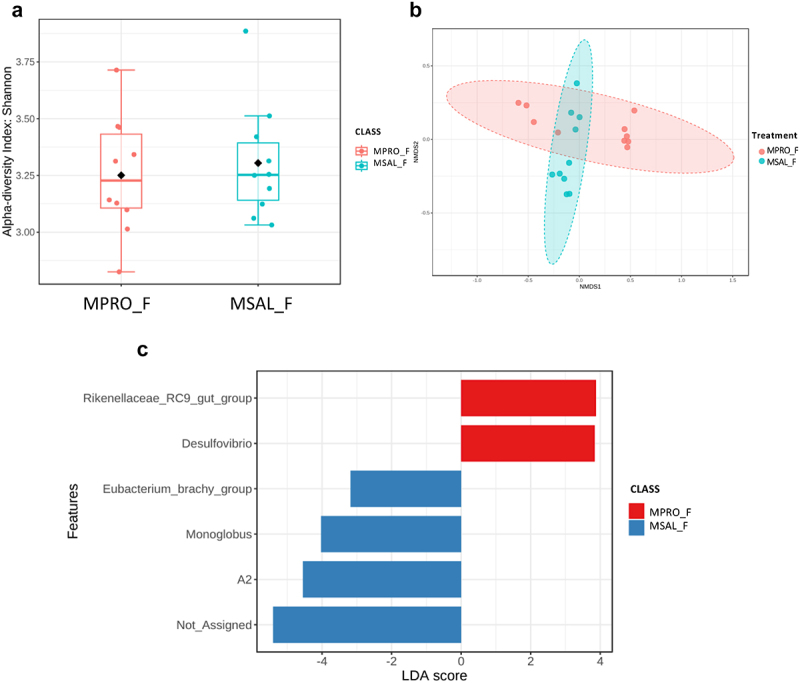
Microbiome analysis of POE female offspring with or without prenatal exposure to probiotics (MPRO_F vs. MSAL_F) (*n* = 10 per group in all experiments). (a) α-diversity index represented using Shannon metric demonstrated no significant differences in microbial richness. (b) β-diversity analysis represented using the Bray-Curtis metric indicated distinct clustering in the microbial composition (*p* = 0.02). (c) LefSe analysis of bacterial taxa at the genus level.

**Figure 8.**
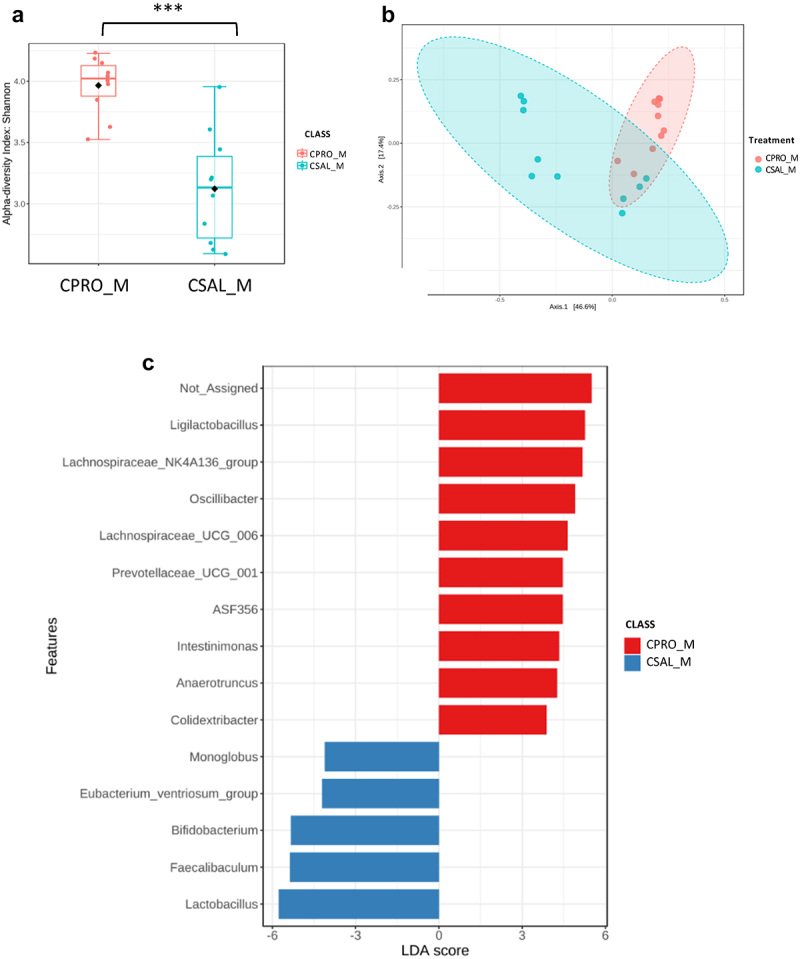
Microbiome analysis of control male offspring with or without prenatal exposure to probiotics (CPRO_M vs. CSAL_M) (*n* = 10 per group in all experiments). (a) α-diversity index represented using Shannon metric demonstrated significant differences in microbial richness. (b) β-diversity analysis represented using the Bray-Curtis metric indicated distinct clustering in the microbial composition (*p* = 0.001). (c) LEfSe analysis of bacterial taxa at the genus level.

**Figure 9.**
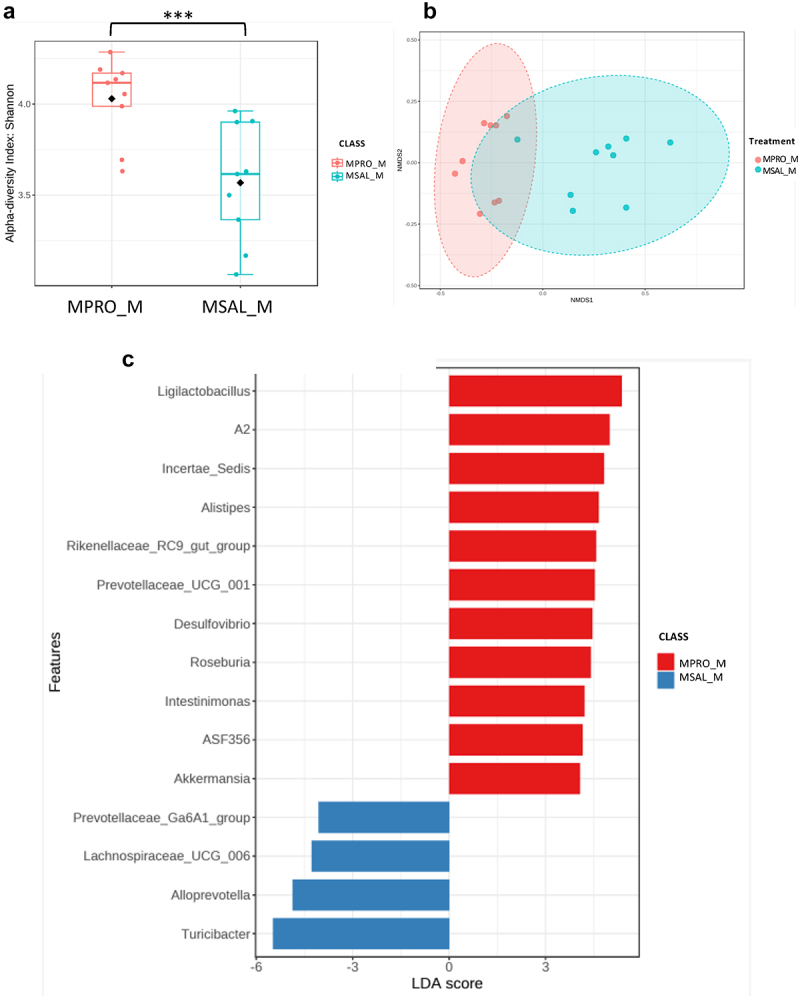
Microbiome analysis of POE male offspring with or without prenatal exposure to probiotics (MPRO_M vs. MSAL_M) (*n* = 10 per group in all experiments). (a) α-diversity index represented using Shannon metric demonstrated significant differences in microbial richness. (b) β-diversity analysis represented using the Bray-Curtis metric indicated distinct clustering in the microbial composition (*p* = 0.001). (c) LEfSe analysis of bacterial taxa at the genus level.

To determine the impact of probiotic supplementation on the gut microbiome, 16s rRNA sequencing was again used. The addition of probiotics resulted in no significant differences in the α-diversity of control females (Figure 6a), but significant differences were observed in β-diversity (Figure 6b). Bacterial taxa such as *Lactobacillus*, *Ligilactobacillus*, and *Lachnoclostridium* were abundant in CPRO_F, whereas CSAL_F had an abundance of different taxa including *Bacteroides, Akkermansia*, and *Turicibacter* (Figure 6c). Probiotic intervention with opioid exposure (MPRO_F) showed no significant differences in α-diversity compared to opioids alone (MSAL_F), but did reveal significant differences in β-diversity (Figure 7a, b). Only two bacterial taxa were enriched in MPRO_F, while three known taxa were abundant in MSAL_F when comparing both groups (Figure 7c). In males, probiotic intervention in control male offspring (CPRO_M) created significant differences in microbial composition and resulted in an abundance of bacterial taxa such as *Ligilactobacillus, Lachnospiraceae* (NK4A136 and UCG-006), and *ASF356* relative to CSAL_M (Figure 8). In comparing MSAL_M and MPRO_M, significant differences were observed in both α and β-diversity (Figure 9a,b). An abundance of several taxa including *Ligilactobaciilus, Akkermansia*, and *Intestimonas* was noted in MPRO male offspring (Figure 9c). Together, the data suggests that maternal probiotic supplementation modifies the gut microbiota of both male and female offspring.

*** *p* ≤ 0.005.

*** *p* ≤ 0.005.

### Sex-based differences in the transcriptomics of the prefrontal cortex

The prefrontal cortex (PFC) is responsible for executive functions and pain processing. Alterations in gene expression, neurotransmitter release, and neuroinflammation during pain are all observed in the PFC, reflecting its connections to other brain regions.^44^ Accordingly, RNA sequencing of the PFC was used to identify altered pathways between offspring that may help explain behavioral differences.

**Figure 10.**
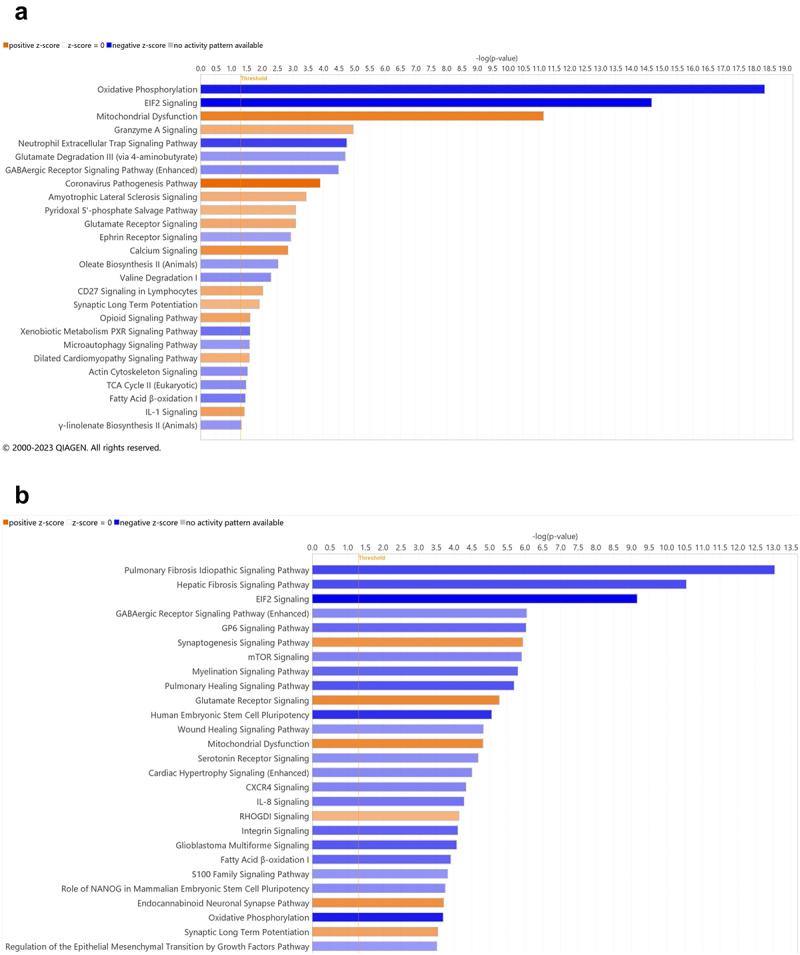
RNA sequencing of offspring (MSAL vs. CSAL) (*n* = 6 per group). (a) Canonical pathways of female offspring (MSAL_F v CSAL_F). (b) Canonical pathways of male offspring (MSAL_M v CSAL_M).

Compared to CSAL_F, MSAL_F exhibited increases in several pathways (Figure 10a). Among these were the opioid signaling pathway (Figure 10a) and pathways related to neurological diseases (Supplemental Figure 2A), predicting increases in motor dysfunction or movement disorder, paralysis, gliosis, and astrocytosis in MSAL_F. Decreased nervous system development was also predicted, as demonstrated by reduced neuroglia quality, neuronal density, dendritic growth/branching, and central nervous system myelination. Additionally, there were predicted increases in several behavioral pathways, including those related to contextual fear memory and psychological disorders. While no sub-pathways reached statistical significance, pathways related to hyperactive behavior and behavioral deficits showed trends toward significance.

Similarly, MSAL_M demonstrated differences in several pathways compared to CSAL_M (Figure 10b), including those related to nervous system development, neurological diseases, behavior, and psychological disorders. However, unlike in females, the opioid signaling pathway was not altered (Figure 10b and Supplemental Figure 2B). More specifically, MSAL_M showed predicted decreases in neuroglial proliferation and quantity among other changes. In terms of neurological diseases, several sub-pathways were predicted to be altered, most notably an increase in sensory loss and cardiomyopathy.

## Discussion

The present study investigates sex-based differences in thermal pain sensitivity and gut microbiome composition following prenatal opioid exposure (POE), with a particular focus on the modulatory potential of maternal probiotic administration. Our findings demonstrate that maternal probiotic supplementation modulates neonatal gut microbiome composition and pain responses in a sex-dependent manner. Specifically, probiotic treatment mitigated adverse outcomes in opioid-exposed offspring by fostering distinct changes in microbial profiles and nociceptive responses according to sex.

Human studies have indicated that signs and symptoms of NOWS, including respiratory distress and hyperreflexia, tend to be more severe in male infants.^45,46^ Our findings reinforce this male susceptibility, as male offspring prenatally exposed to opioids showed hypersensitivity to thermal pain at both 3-weeks and 5-weeks-old. Microbiome analyses of opioid-exposed male offspring completed at 5-weeks-old identified a notable enrichment of bacterial taxa such as *Tyzzerella* (from the *Lachnospiraceae* family), which has been associated with high cardiovascular risk profiles,^47,48^ and *Alloprevotella*, previously linked to pro-inflammatory markers.^49^ Additionally, gene expression analyses in the prefrontal cortex revealed sex-specific differences related to neural development, behavior, and susceptibility to neurological disorders. Notably, female offspring exposed to opioids showed an increase in the opioid signaling pathway, potentially explaining an opioid-induced analgesic effect absent in males. It is known that opioids bind predominantly to the µ-opioid receptor (MOR) and contribute to physiologic responses, including nociception.^50^ Interestingly, it has been found that the MOR is implicated in the modulation of pain^26^ and is responsible for differences in tail-flick thresholds between test subjects that have been exposed to opioids.^51^ Thus, the increase in the opioid signaling pathway of opioid-exposed females could indicate the analgesic effect of opioids still observed in 5-week-old POE female offspring, which was not observed in males (Figures 1b–d and 5), contributing to their hypersensitivity to thermal pain.

To address these sex differences in pain sensitivity, probiotics were administered to both control and opioid-treated pregnant animals. In male offspring exposed to opioids, probiotic administration led to increased prevalence of beneficial bacterial taxa, including *Ligilactobacillus*, *Alistipes*, *Intestinimonas*, and *Akkermansia*. While the pathogenic or beneficial function of gut microbiota are both context and strain specific, the increase of these bacteria have been associated with health benefits like enhanced gut barrier function and anti-inflammatory effects through metabolite production.^52–56^ Probiotics administered during pregnancy may thus improve neonatal gut microbiome composition, though extending the duration of these effects remain an ongoing goal. At 3 weeks, MPRO_M demonstrated improvement in nociception compared to MSAL_M, with no significant differences compared to CSAL_M and CPRO_M. While the effect weakened with time, probiotic-treated males still showed improved outcomes as there was no significant difference between MPRO_M and CSAL_M (Figure 5b,d).

In contrast, female POE offspring (MSAL_F) displayed hyposensitivity to pain relative to controls (CSAL_F) at both 3 and 5 weeks (Figure 5a,c). The gut microbiome of female POE offspring was enriched with *Lactobacillus*, potentially supporting their resilience against POE-associated changes.^57^ However, other bacterial taxa, such as *Ruminococcus*, was also enriched and has been linked to various intestinal and systemic effects, including associations with inflammatory bowel disease (IBD) and neurological conditions.^58^ Probiotic treatment in control females (CPRO_F) also led to hyposensitivity at 3 weeks compared to controls (CSAL_F), though this effect diminished by 5 weeks, possibly explained by no significant difference in alpha diversity. We hypothesize the combination of the opioid signaling pathway and increased abundance of commensal bacteria contributed to the prolonged hyposensitivity observed in MSAL_F; the absence of this combination in CPRO_F may have contributed to the diminished effect at 5-weeks-old. Probiotics did not significantly impact pain sensitivity in opioid-exposed females (MPRO_F), potentially due to minimal changes in alpha diversity (Figure 7a) and limited differentially enriched bacterial taxa between MSAL_F and MPRO_F (Figure 7c).

Collectively, our findings indicate distinct sex-based responses to POE, particularly in the composition of the gut microbiome and nociceptive response. This study adds to growing literature on sex differences in gut microbial composition^59^ and pharmacokinetics/pharmacodynamics,^60^ which may be influenced by hormonal factors. Male POE offspring exhibited sustained hypersensitivity to thermal pain (Figures 1c,e and 5b,d), while females displayed hyposensitivity at both 3- and 5-weeks of age (Figures 1b,d and 5a,c), likely influenced by opioid signaling pathways^26,50,51^ and microbial profiles dominated by commensals like *Lactobacillus*. Probiotic supplementation shifted the gut microbiome composition in male offspring toward a beneficial profile, improving nociceptive responses in the early postnatal period. In female offspring, maternal administration of probiotics significantly increased the threshold of the control group at 3-weeks-old, but had no significant impact on those that were exposed to opioids in utero. These findings highlight the therapeutic potential of probiotics for alleviating gut dysbiosis and nociceptive hypersensitivity following POE. However, RNA sequencing revealed sex-specific pathways that could negatively impact neurodevelopment, underscoring the complexity of POE’s impact on the gut-brain axis and nociception. For example, RNA sequencing of the prefrontal cortex in MSAL_M predicted an increase in sensory loss and decreased proliferation and quantity of neuroglia. RNA sequencing of female offspring showed those exposed to opioids in utero may have an increased susceptibility to neurological diseases and psychological disorders and decreased nervous system development.

Limitations of this study include translational aspects to human models and the effects of environmental confounders. Future studies should assess whether these changes persist into adulthood and examine the mechanisms underlying sex-based differences in the gut-brain axis. Identifying therapeutic approaches that sustain beneficial microbial and behavioral changes will be crucial for long-term modulation of pain sensitivity.

## Supplementary Material

Supplemental Material

## Data Availability

The authors confirm that the data supporting the findings of this study are available within the article and/or its supplementary materials.
