# Supplementary material for: Probiotic supplementation mitigates sex-dependent nociceptive changes and gut dysbiosis induced by prenatal opioid exposure
Source: Gut Microbes. 2025 Feb 14;17(1):2464942. doi: 10.1080/19490976.2025.2464942 (PMC11834462; doi:10.1080/19490976.2025.2464942)
Supplement: Supplemental Material [file KGMI_A_2464942_SM2358.docx]

**Supplemental Figures**


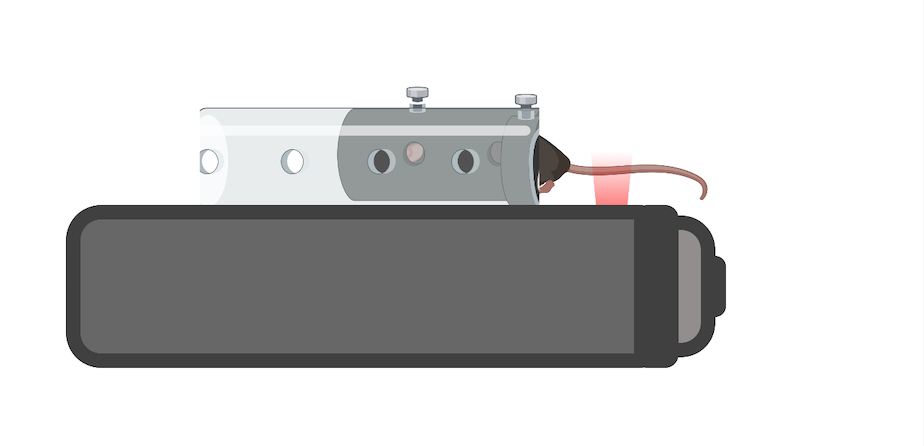


**Supplemental Figure 1.** **Schematic of tail-flick assay used to assess thermal pain sensitivity.** Each mouse was placed in the restrainer, with its tail positioned over a beam of light that activates to generate heat when prompted.


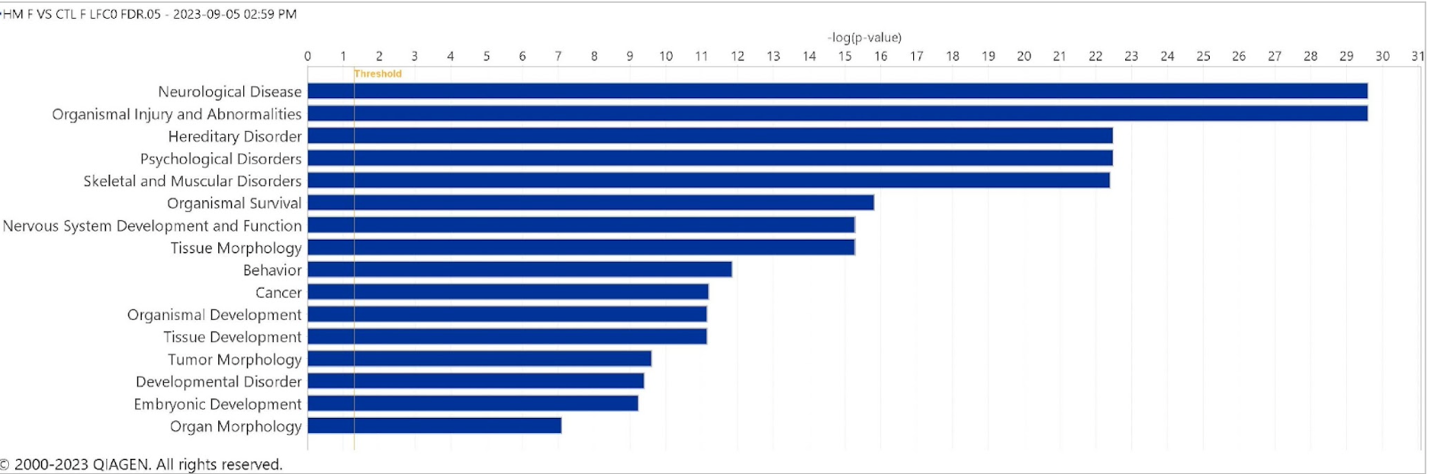
A


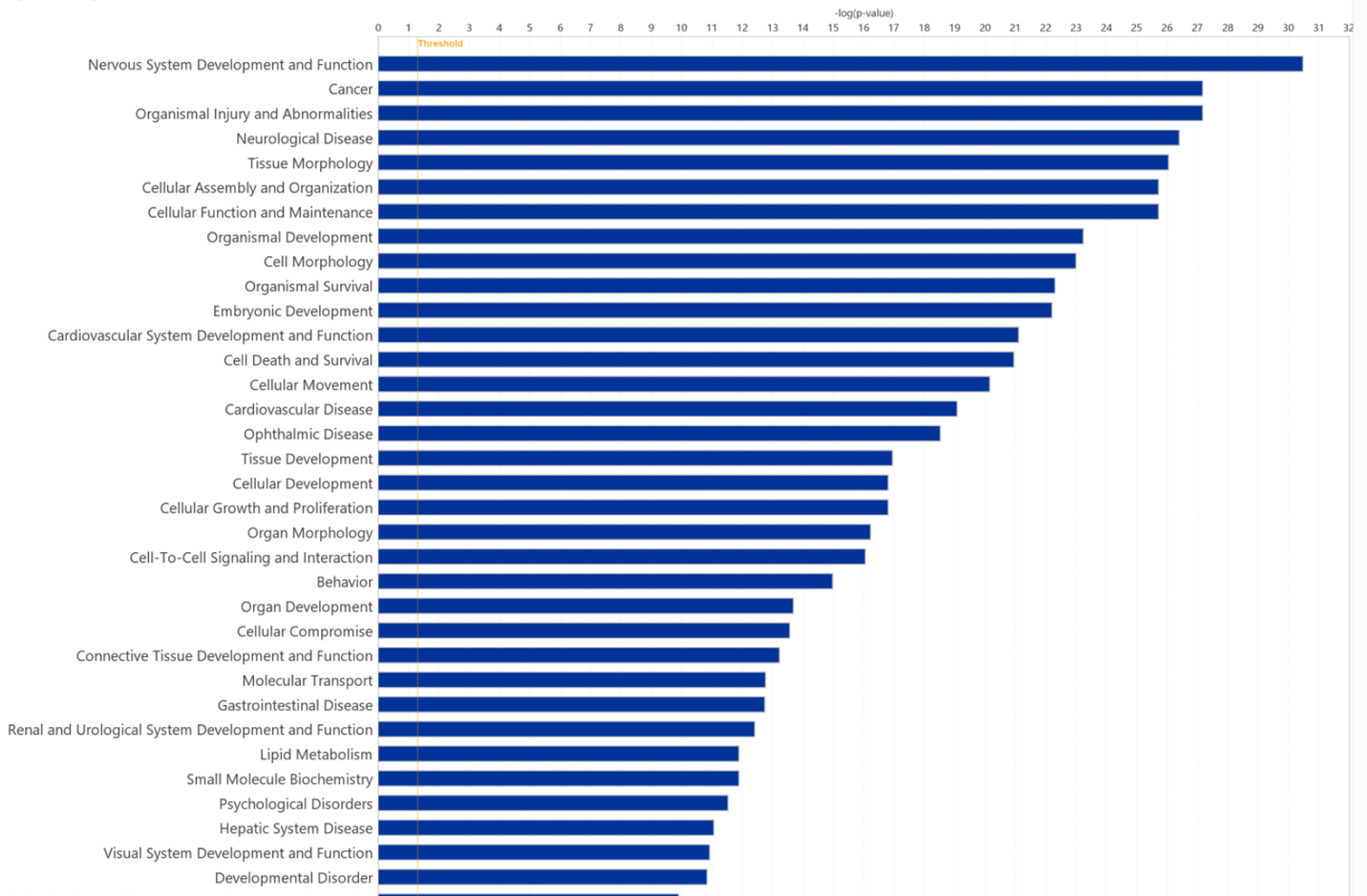
 B

**Supplemental Figure 2.** **Upregulated diseases and functions in opioid-exposed animals relative to controls** (n=6 per group). (A) Upregulated diseases and functions in opioid-exposed female offspring relative to controls (MSAL_F vs. CSAL_F). (B) Upregulated diseases and functions in opioid-exposed male offspring relative to controls (MSAL_M vs. CSAL_M).
